# Pulse consumption trends in the US in the context of 2025–30 Dietary Guidelines for Americans: analyses of NHANES data for 1999–2018

**DOI:** 10.3389/fnut.2025.1638519

**Published:** 2025-08-21

**Authors:** Adam Drewnowski, Catherine Zavela, Vanessa Tran, Greeshma Mallya, Zach Conrad

**Affiliations:** ^1^Center for Public Health Nutrition, University of Washington, Seattle, WA, United States; ^2^Food Systems, Nutrition and Health Program, School of Public Health, University of Washington, Seattle, WA, United States; ^3^Global Research Institute, William & Mary, Williamsburg, VA, United States; ^4^Department of Health Sciences, William & Mary, Williamsburg, VA, United States

**Keywords:** pulses, NHANES 1999–2018, education, incomes, Mexican Americans, 2025–30 Dietary Guidelines Advisory Committee (DGAC), plant protein

## Abstract

**Background:**

The 2025–30 US Dietary Guidelines Advisory Committee has recommended moving pulses to the Protein Foods Group and listing them ahead of meat, poultry, and eggs. The recommended amounts went up from 1.5 to 2.5 cups/week per 2,000 kcal daily.

**Objective:**

To track temporal trends in pulse consumption in the US from 1999 to 2018 by consumer socio-demographics and by pulse type.

**Methods:**

Dietary intakes data came from 10 cycles of the National Health and Nutrition Examination Survey (NHANES 1999–2018). Adult participants were stratified by sex, age group, income to poverty ratio (IPR), education, and race/ethnicity Pulses were separated into beans, chickpeas, peas, and lentils. Analyses were conducted for the whole sample (*n* = 48,738) and for pulse consumers (*n* = 9,186). Trend analyses were based on linear regression models.

**Results:**

Across all years tested, only 17.2% of NHANES participants consumed any pulses on a given day. Mean prevalence was highest among Mexican Americans (33.3%) and lowest among non-Hispanic Black groups (12.0%). Consumption prevalence dropped after 1999–2000 but recovered after 2007–08. Mean consumption of cooked pulses was 0.39 oz/day overall and 2.26 oz/day among consumers. Higher amounts were consumed by Mexican Americans, men, and groups with lower education and incomes. In 2017–2018, 19.9% of the population consumed 1.5 cups/week of cooked pulses and 10.9% consumed 2.5 cups/week. Beans were the most consumed pulse followed by lentils, chickpeas, and peas. Unlike beans, both lentils and chickpeas were associated with higher socioeconomic status.

**Conclusion:**

Other than among Mexican Americans, pulse consumption in the US was low and was associated with lower education and incomes. However, pulse consumers consumed substantial amounts. Lentils and chickpeas may represent opportunities for increasing pulse consumption. The present findings are relevant to the implementation of the 2025–30 Dietary Guidelines for Americans and can help target the promotion of pulses among population subgroups.

## 1 Introduction

Beans, lentils, chickpeas, and dried peas, collectively known as pulses, are dietary staples in many parts of the world (1). Pulses are inexpensive sources of plant protein, dietary fiber, and multiple micronutrients (1, 2). Compared to many sources of animal protein, pulses are more affordable and more environmentally friendly (2). Past analyses (2) have pointed to a lower water footprint of pulses and lower greenhouse gas emissions.

To promote plant-forward diets in the US, the 2025–2030 Dietary Guidelines for Americans Advisory Committee (DGAC) suggested moving beans, peas, and lentils from the vegetable food group to the Protein Foods Group (3) and listing them above meat, eggs, and dairy as potentially healthier sources of plant protein (4). The DGAC also recommended increasing the consumption of cooked pulses from the current guideline of 1.5–2.5 cups/week, based on a 2,000 kcal/day diet (3).

Potential barriers to implementing the DGA can be identified by examining existing consumption patterns. However, data on pulse consumption trends in the US are scarce (5–7). A previous study (5), based on six cycles of the nationally representative National Health and Nutrition Examination Survey (NHANES 2003–14), estimated population daily intakes of cooked pulses at about 20 g/day or just over $\frac{1}{2}$ cup/week. Daily intakes of pulse consumers were estimated at 70.9 g/day, which is about 2.0 cups per week (5). Strong variations by education and race/ethnicity were also observed (5). Past analyses of NHANES data have also pointed to the high consumption of pulses and pulse-based mixed dishes by Hispanic groups (2), notably by Mexican-Americans. Past analyses of NHANES 2017–18 data placed the consumption of all legumes at 0.48 oz/day for males and females aged >20 year (8).

Very few studies have explored social gradients in pulse consumption in the US over a 20-year period. The present analysis of pulse consumption trends by age, sex, socioeconomic status, and race/ethnicity can help identify potential ways to implement the 2025–30 Dietary Guidelines for Americans (DGA) (9). Novel analyses by pulse type (beans, chickpeas, lentils, and peas) can help assess the appeal of different pulses across socio-demographic groups. The present study used 10 cycles of NHANES for the years 1999–2018 separated pulses by type, and tracked time trends in consumption of cooked pulses across diverse socio-demographic groups. The findings have implications for dietary guidance and the promotion of plant based diets in the US.

## 2 Methods

### 2.1 Dietary and sociodemographic data

Dietary intakes data came from 10 cycles of the National Health and Nutrition Examination Survey (NHANES 1999–2018). NHANES data were collected by the National Center for Health Statistics (NCHS) using a clustered, stratified, multi-stage sampling design and released in 2 year cycles (10), with ~5,000 participants per cycle (11). Some demographic groups were oversampled to increase reliability and precision for subgroup analysis. The present sample was 48,738 adult participants, aged ≥20 years. The sample was stratified by sex (male, female), age group (20–30, 31–50, 51–70, and >70 years), income to poverty ratio (< 1.85 and >1.85), and education (high school or less, some college or more). Race/ethnicity groups were non-Hispanic White, Non-Hispanic Black, Mexican American, and Other group that included Asians, other Hispanic groups, and multiracial populations.

The income to poverty ratio (IPR) was defined as the ratio of household income to the federal poverty threshold. The poverty threshold, as defined annually by the U.S. Census Bureau, varies by household size and composition. IPR values, calculated by the NCHS, were used to classify participants into income groups. Lower IPR, a social determinant of health, tends to be associated with higher risk of obesity and non-communicable disease.

The dietary intake component of NHANES is known as the What We Eat in America study (12). Trained NCHS interviewers administered 24-h recalls using the computer-assisted Automated Multiple Pass Method to minimize participant burden and increase data reliability and validity (13, 14). Participants also provided information on sociodemographic characteristics, including age, sex, income, education, and race-ethnicity (15). The Institutional Review Board (IRB) approvals for NHANES were obtained by the National Center for Health Statistics (NCHS) (16). NHANES data are publicly available (17). Details for each survey cycle are available online.

### 2.2 Estimating daily intake of pulses: dry beans, chickpeas, peas, and lentils

Data from the 1st day of dietary recall were used to estimate per capita intake. One day data are sufficient for calculations of group means but do not necessarily reflect usual eating patterns. The NHANES dietary files list the name, description, and amount (gram weight) of each food consumed. Each food had an 8-digit food code based on the predominant ingredient in that food, using the coding scheme established by the USDA Food and Nutrient Database for Dietary Studies (FNDDS) (18). A two-step procedure was used to estimate the amount of each type of pulse (dry beans, chickpeas, peas, and lentils) consumed by each participant. First, all foods that contained pulses (as determined based on their text descriptions) were flagged. This resulted in 339 pulse-containing foods (beans = 279 foods, chickpeas = 20 foods, lentils = 17 foods, and peas = 23 foods). Second, the amount of pulses present in each food was estimated using the USDA Food Pattern Equivalents Database (FPED) (19). The USDA FPED lists the amount (in oz. Equivalents) of legumes present in 100 g of each food consumed by NHANES participants. The key steps in the conversion, as documented by the USDA, were as follows. First, each FNDDS code was linked by the FPED data file (Food Pyramid Equivalents Database) to a recipe file. Soups and mixed meals were disaggregated into components to estimate the amount of pulses. Beans, lentils, chickpeas, and dried peas counted toward the legumes component. The amount of pulses was converted to ounce equivalents (oz eq) of legumes using USDA Food Pattern conversion factors. The standard conversion is $\frac{1}{4}$ cup of cooked pulses to 1 oz equivalent, based on the contribution to the protein food group.

### 2.3 Statistical analyses

Pulse consumers and non-consumers were identified based on consumption of >0 oz equivalents of pulses on day 1 of dietary recall. Differences in socio-demographic characteristics between consumers and non-consumers were tested using Pearson's chi-squared statistic with significance set as *p* < 0.05. Among consumers, temporal trends in pulse intake by sociodemographic characteristic were tested using linear regression models adjusted for total energy intakes (kcal), with significance set as *p* < 0.05. A Bonferroni adjustment was used to correct for comparisons among multiple means. NHANES design variables and survey weights were used to account for the complex sampling design and to produce nationally representative estimates. Analyses used STATA statistical software 16.1 (20).

## 3 Results

### 3.1 Participant characteristics

Table 1 shows the principal characteristics of pulse consumers (*n* = 9,186) and non-consumers (*n* = 39,552). Differences between consumers and non-consumers were tested using Pearson chi-squared statistic. There were significant effects of age, sex, and race/ethnicity (*p* < 0.01). No effects of income or education were observed.

**Table 1:** Characteristics of study participants, 1999–2018 (*n* = 48,738).

**Characteristic**	**Pulse consumers (*****n*** = **9,186)**^**a**^			**Pulse non-consumers (*****n*** = **39,552)** ^**a**^			**P^b^**
	***n***	**% (95% CI)**		***n***	**% (95% CI)**		
**Age, year**							< 0.001
20–30	1,687	20.5	(19.2–21.9)	7,692	21.0	(20.1–21.9)	
31–50	3,369	41.3	(39.4–43.3)	12,954	36.5	(35.6–37.5)	
51–70	2,978	29.8	(28.1–31.5)	12,236	30.7	(29.7–31.6)	
70+	1,152	8.4	(7.6–9.3)	6,670	11.8	(11.3–12.4)	
**Gender**							< 0.001
Male	4,622	50.6	(49.4–51.9)	18,885	47.6	(47–48.2)	
Female	4,564	49.4	(48.1–50.6)	20,667	52.4	(51.8–53)	
**Income/poverty ratio** ^b^							0.838
≤ 1.85	3,892	32.9	(30.8–35.1)	15,857	32.7	(31.4–34.1)	
1.86+	4,422	67.1	(64.9–69.2)	20,438	67.3	(65.9–68.6)	
**Education**							0.863
High school	4,933	41.2	(39.2–43.2)	19,445	41.3	(39.9–42.8)	
Some college	4,239	58.8	(56.8–60.8)	20,060	58.7	(57.2–60.1)	
**Race-ethnicity**							< 0.001
Non-Hispanic White	3,102	61.0	(58.2–63.7)	18,763	69.9	(67.9–71.9)	
Non-Hispanic Black	1,181	7.8	(6.9–8.9)	9,014	12.0	(10.8–13.2)	
Mexican-American	2,993	15.8	(13.8–17.9)	5,563	6.6	(5.8–7.6)	
Other^c^	1,910	15.4	(13.8–17.2)	6,212	11.5	(10.6–12.5)	

^a^Sample sizes are unweighted.

^b^Incomes data based on 8,314 consumers and 36,295 non-consumers.

^c^Includes other Hispanic, non-Hispanic Asian, and multi-racial.

### 3.2 Consumption prevalence time trends

**Figure 1:**
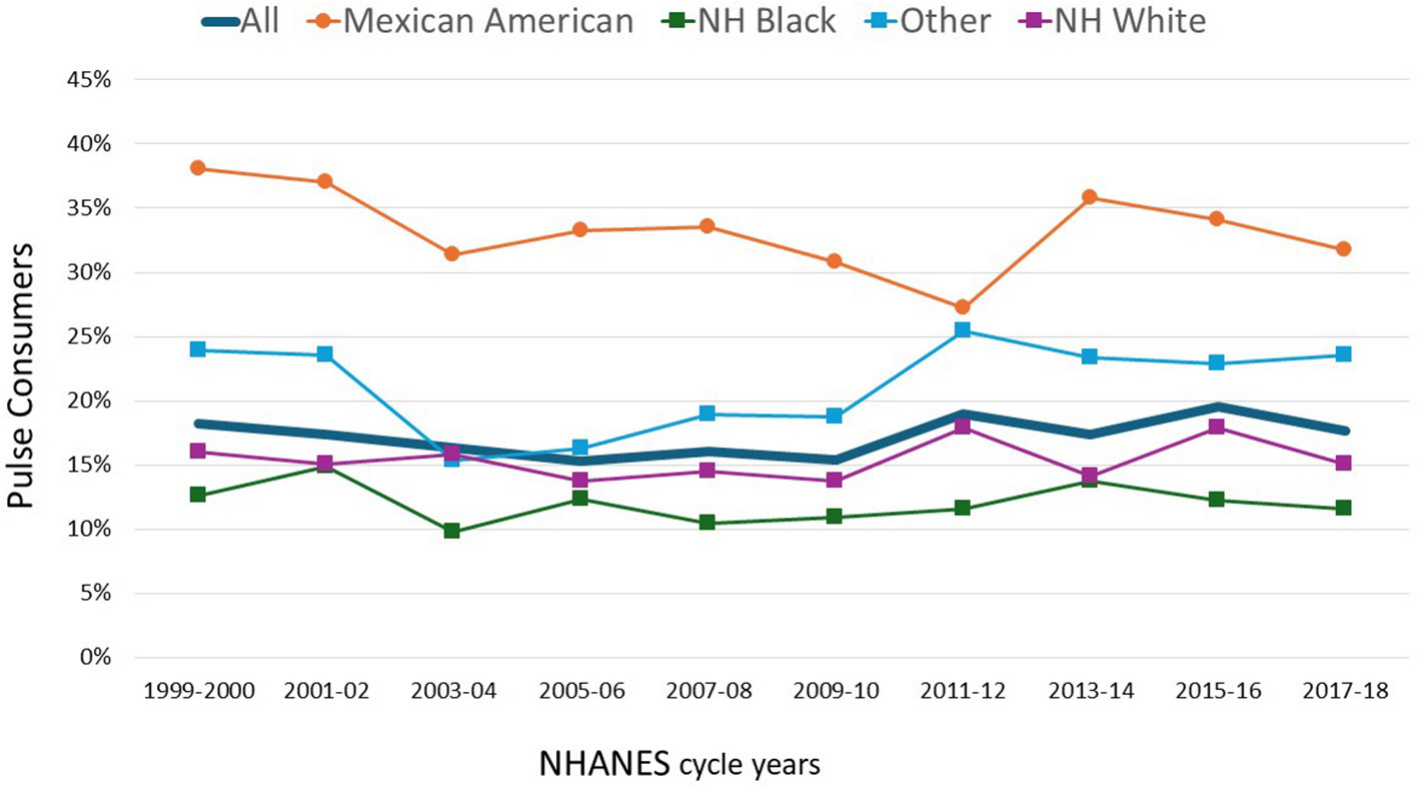
Percentage of NHANES participants who consumed cooked pulses on the 1st day of NHANES surveys 1999–2018 by race/ethnicity (*n* = 48,738).

Figure 1 shows time trends in the proportion of pulse consumers. Time trends for the US population show that there was little change from 1999 to 2018 (*p* for trend 0.155). The significant drop in prevalence from 1999 to 2006 (*p* for trend 0.037) was followed by a significant increase between 2006 and 2008 (*p* for trend 0.009).

Figure 1 also shows significant differences in consumption prevalence by race/ethnicity. Mexican Americans were most likely to consume pulses, followed by the Other groups. The group least likely to consume pulses were non-Hispanic White and non-Hispanic Black groups. The prevalence of consumers in the Other group has been increasing since 2003–2004. Data for Figure 1, along with statistics are shown in Supplementary Table S1.

**Figure 2:**
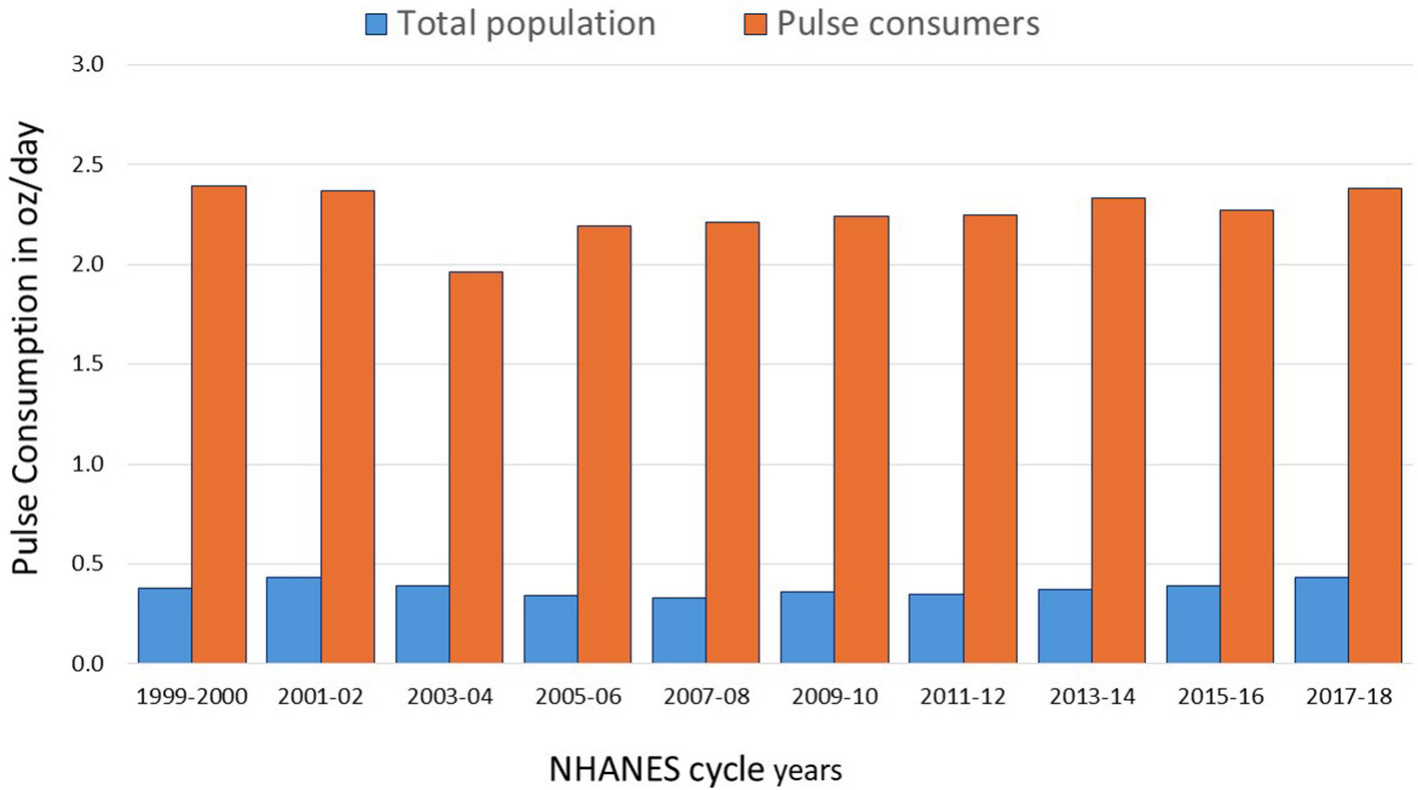
Amounts of cooked pulses consumed (oz/day) by total population and by pulse consumers.

Figure 2 shows that mean amounts consumed over the 20 period were 0.39 oz/day (12 g/day) or 84 g/week overall. Figure 2 compared estimated amounts for the population and for consumers only. Those NHANES participants who consumed pulses (*N* = 9,186) consumed 2.26 oz/day (68 g/day) or 476 g/week. While the population consumption values were low, the small group of pulse consumers came close to the recommended amounts.

### 3.3 Time trends in population amounts consumed (oz/day) by pulse type

**Figure 3:**
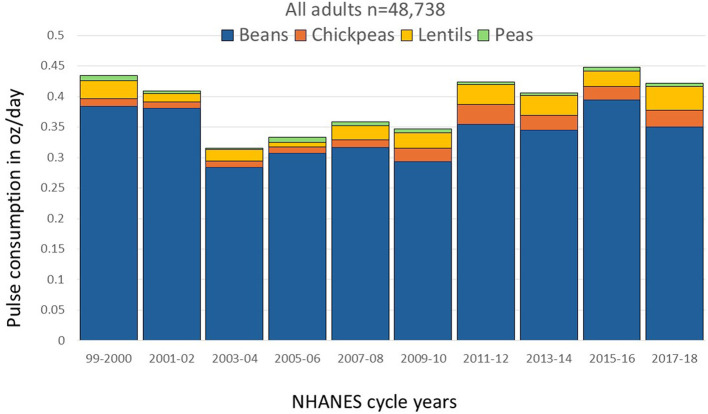
Trends in cooked pulse consumption by pulse type in oz/day among all NHANES participants, 1999–2018.

Time trends in pulse amounts consumed by pulse type are shown in Figure 3. The most consumed pulses were beans, followed by lentils and chickpeas. Peas were the least consumed pulse. The consumption of chickpeas has increased since 1999–2000 (*p* for trend < 0.001). The increase for lentils was not significant (*p* for trend 0.092). Data for Figure 3 are in Supplementary Table S2.

### 3.4 Time trends in population amounts consumed (oz/day) by socio-demographics

**Figure 4:**
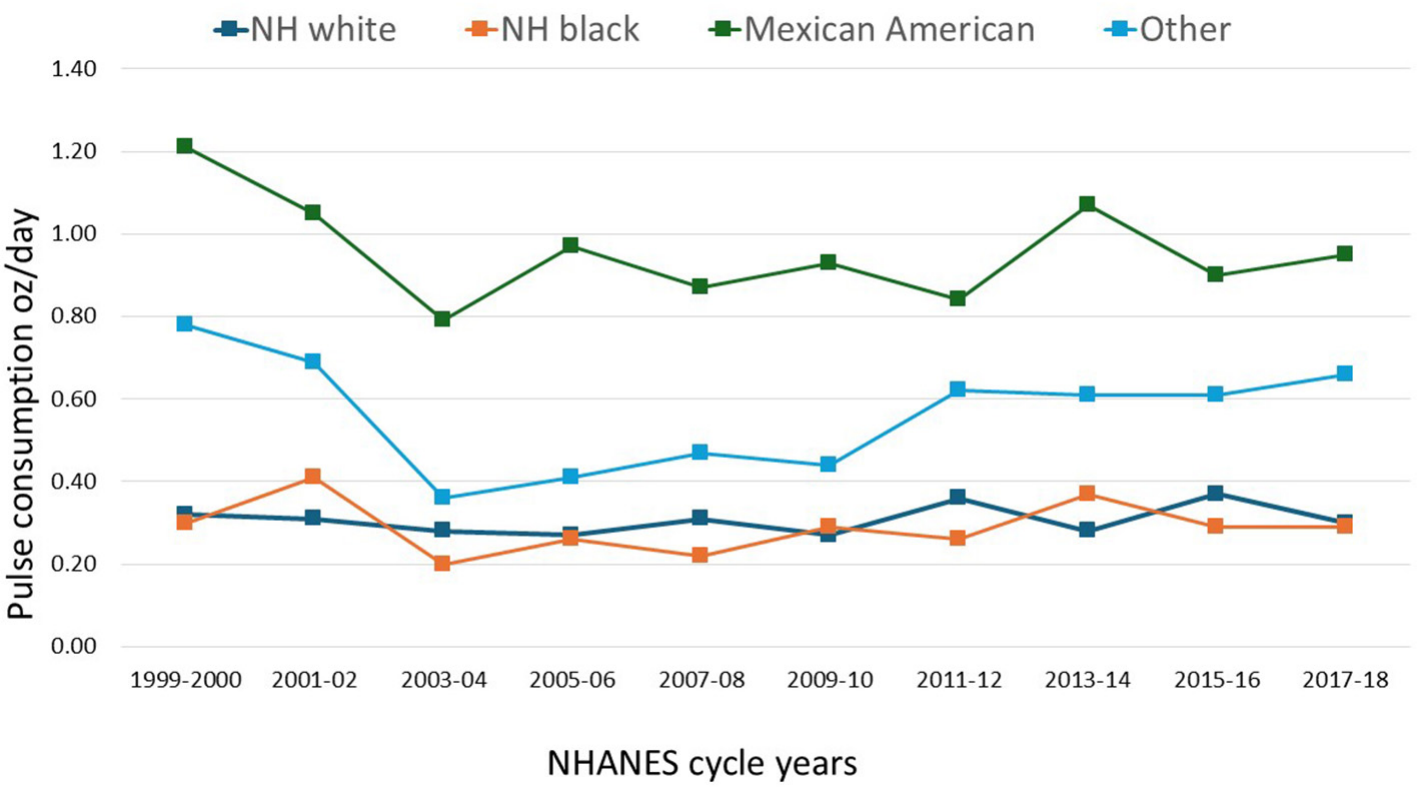
Time trends in the amounts of cooked pulses in oz/day consumed in the total population by race/ethnicity, NHANES 1999–2018 (*n* = 48,738).

Figure 4 summarizes time trends in the amounts of pulses consumed by sociodemographic variables. The highest amounts by far were consumed by Mexican Americans followed by the other group (*p* < 0.001). However, pulse consumption among Mexican Americans has declined since the peak in 1999 (*p* for trend 0.397) with no later increase. The Non-Hispanic White group consumed the lowest among of pulses and no increase with time was observed (*p* for trend 0.415).

**Figure 5:**
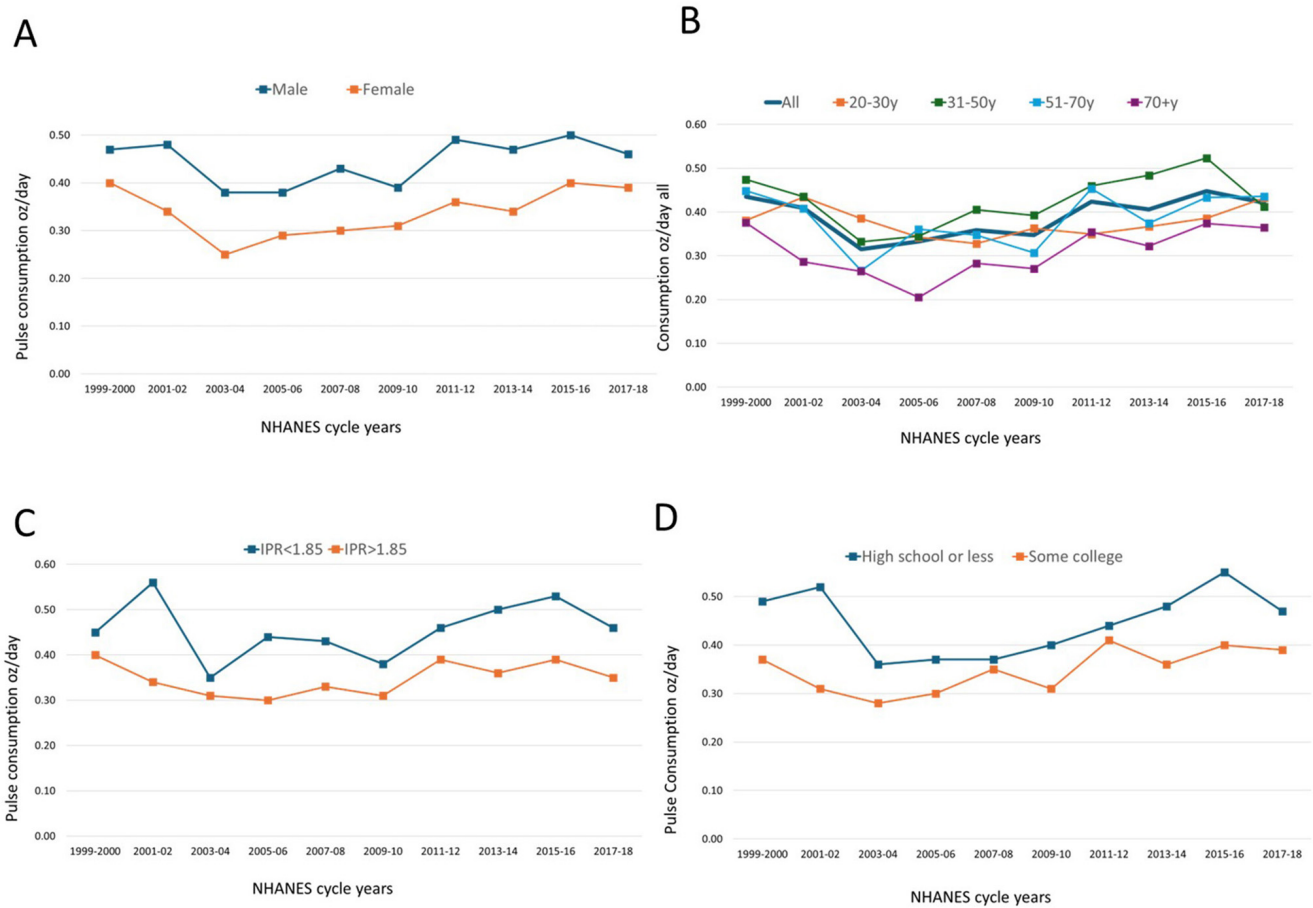
Mean daily consumption of cooked pulses by socio-demographic variables. NHANES 1999–2018. Data are for sex **(A)**, age groups **(B)**, incomes **(C)**, and education **(D)**.

The remaining trends were less pronounced. Figure 5A shows that men consumed higher amounts of pulses than did women. Figure 5B shows that higher amounts of pulses were consumed by younger adults ages 31–50 years. By contrast older adults (aged 70+ year) consumed the lowest amounts of pulses. Figure 5C shows higher consumption of pulses among groups with lower IPR (< 1.86) compared to groups with higher IPR (*p* < 0.001). Figure 5D shows that higher amounts of pulses were consumed by groups with only high school education compared to groups with some college (*p* < 0.001). Data and statistics are in Supplementary Tables S3–S5.

### 3.5 Time trends in consumption for pulse consumers (*n* = 9,186)

Subsequent analyses conducted for pulse consumers only examined time trends and the amounts consumed. Mean consumption over the 20 year period was 2.26 oz/day or 15.8 oz/week, which is 3.96 cups/week. Data for specific pulse types are in Supplementary Table S6.

**Figure 6:**
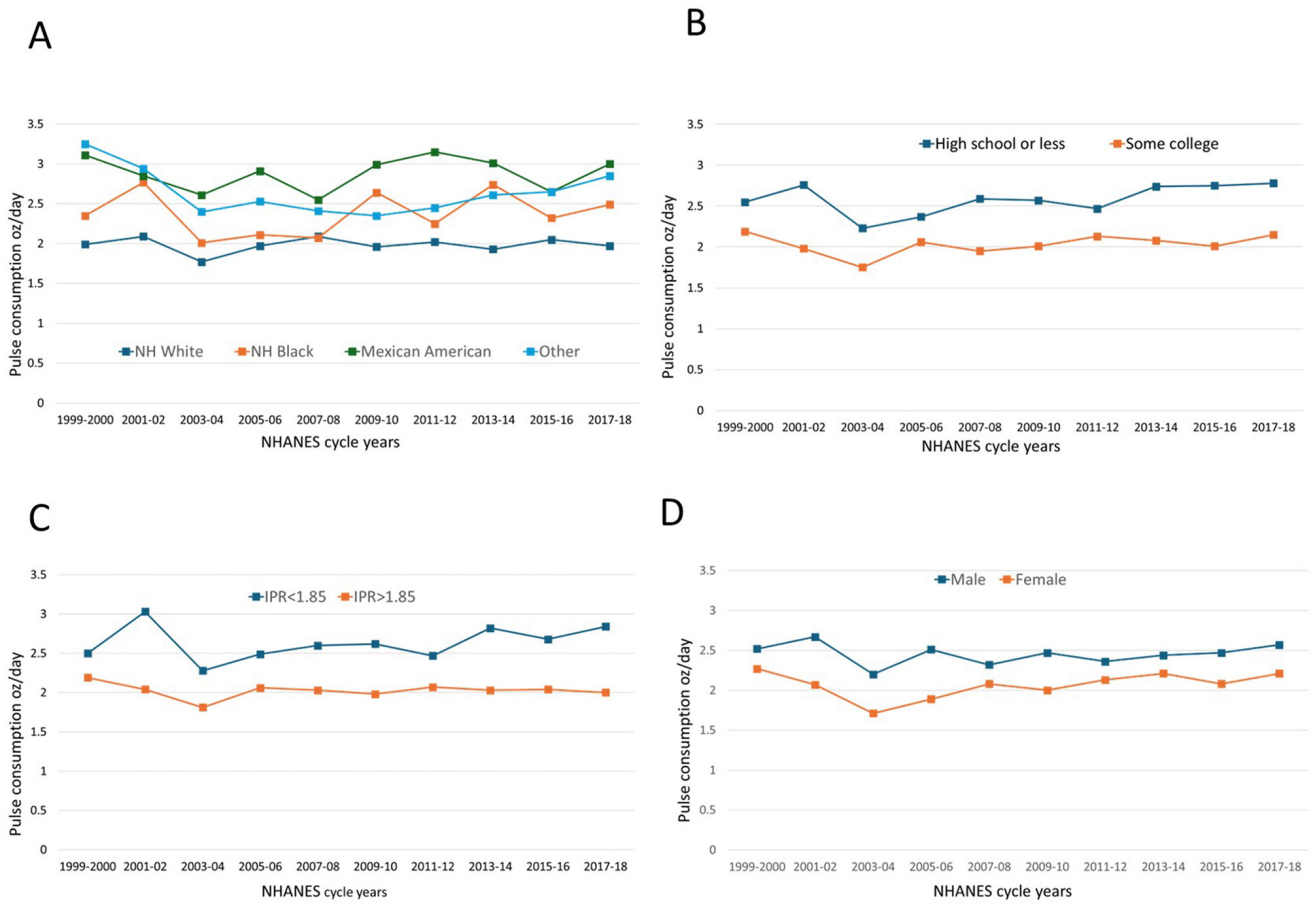
Time trends in cooked pulse consumption (oz/day) for pulse consumers by socio-demographic variables: sex **(A)**, age groups **(B)**, income to poverty ratio **(C)**, and education **(D)**.

Figure 6 shows daily per capita pulse consumption by sociodemographic variables. Figure 6A shows that NHANES participants identifying as Mexican Americans were the largest consumers of pulses, followed by the Non-Hispanic Black group. In 2017–18 mean consumption of pulses in those two groups was ~3.5 oz/day. The Non-Hispanic White group consumed the smallest amounts of pulses compared to the other three groups, below 2 oz/day in some years, and with consumption continuing to decline. Mixed results were obtained for the Other/multiracial group, which includes other Hispanic, Non-Hispanic Asian, and multi-racial individuals. Pulse consumption for this group has risen since 2005–06.

Figure 6B shows that NHANES participants without a high school diploma consumed most pulses. There was some recent growth in pulse consumption for groups with “Some college” The association between lower incomes and higher consumption of pulses is shown in Figure 6C. Consumption for higher IPR groups peaked in 2001–02 but declined in 2003–04 and showed no sign of recovery. By contrast, consumption for lower IPR groups has been increasing since 2003–04.

Time trends in per capita intake of pulses in oz/day by sex are shown in Figure 6D. Men consumed more pulses that did women. The 20-to-30-year age group and the 70+ age group showed the highest consumption of pulses in terms of daily amounts. Both groups showed consumption between 2015 and 2018. However, pulse intake for the 51–70-year age group decreased during the same periods (Figure 6). The corresponding data are in Supplementary Tables S7, S8.

### 3.6 Amounts consumed by income-to-poverty ratio (IPR) and pulse type

**Figure 7:**
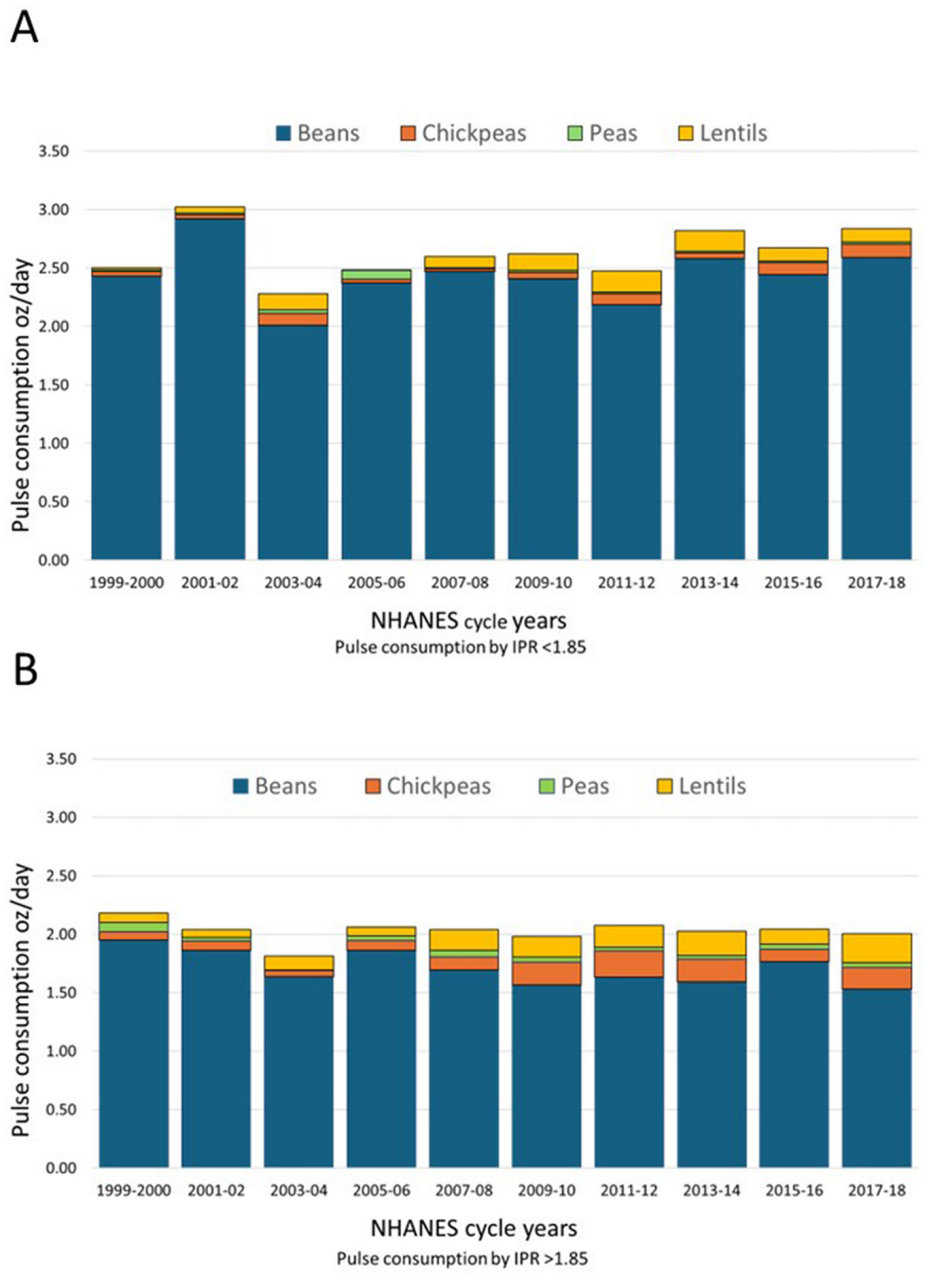
Time trends by income category and pulse type for pulse consumers NHANES 1999–2018. Data are for lower IPR < 1.85 **(A)** and higher IPR (>1.85) **(B)**.

Figure 7A shows that the pulses consumed by lower income groups (IPR < 1.85) were mostly beans. Total consumption reached ~2.5–3.0 oz/day, consistent with previous studies showing that pulse consumption was linked to lower incomes (6, 21). The consumption of lentils, chickpeas, and peas were minimal throughout the study period (22). The temporal pattern revealed notable fluctuation, with consumption declining to ~2.25 oz/day during 2003–04 before recovering to higher levels in subsequent cycles.

Figure 7B shows that higher-income participants consumed less pulses overall, with consumption estimated at 1.5–2.0 oz/day. However, there was much more variety by pulse type: higher income groups consumed more lentils and chickpeas, consistent with some past studies (23, 24). The consumption of peas in this group was still low.

## 4 Discussion

### 4.1 Findings in the context of the DGAC report

The 2025–30 US Dietary Guidelines Advisory Committee (DGAC) recommended moving beans, peas, and lentils to the Protein Foods Group in order to encourage more plant-based diets. The DGAC proposed reorganizing the order of the Protein Foods Group to list beans, peas, and lentils first, followed by nuts, seeds, and soy products, then seafood, and finally meats, poultry, and eggs (3). The DGAC also recommended increasing pulse consumption from 1.5 to 2.5 cups/week on a population-wide basis (3).

That would require a major increase in pulses consumption relative to current amounts. The mean amount consumed in the latest NHANES cycle (2017–18) was estimated at 0.39 oz/day (12 g/day). This value is lower than the previous estimates (5) from earlier NHANES cycles (20 g/day) and falls well-short of both current and proposed recommendations. Whereas, the current consumption of cooked pulses is below $\frac{1}{2}$ cups per week, the DGAC proposed amount is 2.5 cups/week, requiring major changes in dietary habits on the population level.

The proposed placing of pulses in the USDA Protein Foods Group also requires careful consideration. One cup of cooked pulses is estimated to weigh between 170 and 200 g depending on pulse type. The USDA's protein equivalency charts (4) and the Food Buying Guide (25) specify that $\frac{1}{4}$ cup of cooked and drained beans, peas or lentils counts as 1 oz-equivalent in the Protein Foods Group (2). That amount of pulses is supposed to provide ~7 g of protein. In reality $\frac{1}{4}$ cup of cooked pulses does not provide as much protein as 1 oz of chicken or red meat (26).

The present data pointed to a drop in pulse consumption occurring around year 2000 that has been followed by a recovery. While reasons for the drop are unclear, studies point to increased acculturation of Latino and Asian immigrant groups and the adoption of Western dietary patterns by younger age groups (27, 28). Conversely, the more recent recovery may have been driven by cultural and economic forces, including food price inflation and a growing interest in plant-based proteins.

### 4.2 Pulse consumption by socio-demographic variables

Present analyses of NHANES data for 1999–2018 showed that only 17.2% of adult NHANES participants consumed any cooked pulses on a given day averaged across the 20-year period. Most likely to consume pulses were Mexican-Americans (33.3% prevalence), consistent with other analyses of NHANES data (5). Least likely to consume pulses was the non-Hispanic Black group (12.0%). In previous studies (5, 6), consumers of dry beans, peas, and lentils were more likely to be Mexican-Americans and other Hispanic groups. Less likely to consume pulses were non-Hispanic White and non-Hispanic Black groups (7). In past analyses of the 2011–2014 NHANES database (7), consumers of dry beans were predominantly Mexican Americans and groups of Hispanic descent.

When it came to the amounts consumed, Mexican-Americans consumed the most pulses (mainly beans), while the Non-Hispanic White group consumed the least (29). There are several reasons for this. Beans are a staple food in traditional Mexican cuisine, often served daily in meals as refried beans, soups, stews, burritos and tacos. Culinary traditions are passed through generations and persist after migration. Pulses are inexpensive, shelf-stable and can be bought in bulk, dried, or canned. Pulses can be a desirable food for diverse families across income levels.

Incomes are one driver of food choice. The present analyses showed that higher pulse consumption was associated with lower education and lower incomes. NHANES participants who completed only high school or the equivalent consumed the most pulses. A similar social gradient was observed for lower incomes. Those data are consistent with past studies (6, 21) showing that pulse consumption was higher among groups with lower education and lower IPR. However, in one study, legume consumption was not affected by household incomes (7).

The present study introduced a novel separation of pulses by pulse type. Consumers of beans were distinct from consumers of lentils and chickpeas. Those data are consistent with studies showing that Hispanic groups consumed mostly beans and only rarely lentils or dried peas (22). By contrast, the Asian group was more likely to consume lentils and dried peas, in line with the dietary patterns of South Asia (22). The consumption of chickpeas in the present analyses was linked to higher education and incomes, consistent with some past studies conducted in the UK (23) and in the US (24). No effect of sex on amounts consumed was observed in analyses of NHANES 1999–2002 (21) for dry beans and peas or for lentils in NHANES 2017–2018 (6).

### 4.3 How does US pulse consumption compare to other countries?

In 2016, the United Nations FAO launched the International Year of Pulses to uplift pulses and spread awareness about their environmental and health benefits (30). The FAO later declared February 10th as World Pulses Day in 2019 to encourage countries to host events and share messages about the importance of pulses (31). The European Union has launched several initiatives to promote pulses, particularly in agriculture. For example, the presently ongoing LEGUMINOSE project encourages food producers to implement intercropping systems with legumes to improve soil health (32). The Transition Paths to Sustainable Legume-based Systems in Europe (TRUE) project (33), had a similar focus. TRUE aimed to decrease the EU's reliance on imported soy products and synthetic nitrogen fertilizers by increasing “sustainable legume cultivation and consumption across Europe” (33).

Even so, pulse consumption in high-income countries remains low (34). Most of the protein in the typical US and European Union (EU) diets comes from meat, poultry, eggs, and dairy (35), with plant proteins accounting for only about 35% of total dietary protein (36). First, beans were indeed the most consumed pulse in Europe (3,635). In the UK, the proportion of chickpea consumers increased from 6.1 to 12.3% from 2009 to 2019 (23), same as in the US. Chickpea consumers were more likely to be women (23). Women in Poland reported consuming more legumes than did men (36). High consumption of pulses was observed among young Portuguese adults (aged 21–28 years) (37) who were interested in replacing animal protein with pulses. Older adults in Finland (37) were more likely to know how to cook pulses.

According to the Food and Agriculture Organization of the United Nations (FAO), pulse consumption is highest in lower- and middle-income countries (38). India is the largest consumer of pulses, accounting for 27% of the world's total pulse consumption, and is followed by Brazil, Egypt, Turkey, Mexico, Argentina, and China (39, 40). Vegetarian dishes such as spicy lentil- and chickpea-based stews and curries, chickpea hummus, and bean-based dishes are common in South Asia, Turkey, the Middle East and parts of Africa (41). Brazil, another leading consumer of pulses, features black beans (along with pork and beef) in feijoada, a national dish (41). Black beans and pinto beans are the main pulses in Mexico, served in soups, stews, and dishes, then often combined with rice and meat (42). Based on FAO food balance sheets, the consumption of dry pulses in Mexico (mostly beans) was estimated at 12.6 kg/capita/year (42).

Loss adjusted food availability data collected by the USDA (8) has estimated population-wide per capita consumption of dry beans and lentils at 1.4 oz eq/week (43). Pulses triple in weight when cooked, meaning that 2.5 cups of cooked pulses (about 500 g) is equivalent to about 167 g of dry pulses. The DGAC recommendation of 8.6 kg/capita/year would place the ideal US consumption below Mexico but above both Paraguay and Peru (44).

### 4.4 Limitations and strengths

The present study was based on the 1st day of dietary recalls in NHANES that are not necessarily reflective of habitual eating patterns. The identification of cooked pulses relied on the USDA FPED data file. Pulse types were assigned based on product descriptions and recipes. It may be that some pulses contained in mixed dishes were omitted from the present analyses. Even with 10 cycles of NHANES spanning 20 years, there was not sufficient power to conduct separate analysis of time trends by pulse type (beans, chickpeas, lentils, and peas) by age group, income, education, and race/ethnicity. We would have liked to explore the likely impact on pulse consumption of age group, acculturation, and pulse type (lentils, chickpeas). The strength of the present analyses was the use of successive NHANES cycles. The nationally representative NHANES survey are the principal means of dietary surveillance and provide support for food policies and programs in the US. Based on current estimates, the multi-year NHANES sample was representative of 217,265,070 adults in the US.

### 4.5 Implementing Dietary Guidelines for Americans 2025–30

The present findings illustrate the complexity and contradictions underlying pulse consumption trends in the US. First, pulses were consumed infrequently, by fewer than one in five NHANES participants on a given day. On the other hand, those NHANES participants who were pulse consumers did consume large amounts of pulses estimated at 2.26 oz/day or 3.96 cups/week. Second, pulse consumption was associated with lower education and incomes. The very affordability of pulses may have created a stigmatizing association with poverty (45). Pulse consumers may have consumed pulses by necessity rather than by choice.

The present data provide an insight into pulse consumption across diverse population subgroups. Specifically, we found socio-economic differences in consumption trends by pulse type. Although beans remained the most popular pulse by far, there was a trend toward higher consumption of lentils and chickpeas by groups of higher education and incomes. Also consuming more chickpeas and lentils was the Other ethnic group, which included South and South East Asians. Those groups could be potential targets for the promotion of pulse consumption in the 2025–30 issue of the Dietary Guidelines.

Two strategies for implementing DGA 2025–30 are suggested. Given that existing pulse consumers already came close to the DGAC recommended goal, one strategy would be to appeal to non-consumers by broadening the appeal of pulses among more diverse population subgroups. The primary consumers of pulses are likely consuming them in traditional, affordable, and minimally processed forms. Emphasizing these culturally familiar uses of beans, chickpeas, and lentils across diverse population subgroups would support equity in the implementation of the DGA. Pulses can be added easily to homemade soups, salads, spreads, and smoothies to increase nutrient density (46). In particular, the promotion of chickpeas and lentils could be targeted at higher income consumers and ethnic groups with fewer cultural or structural barriers regarding plant based foods.

The popularity of plant-based diets is reported to be growing among younger and higher-income Americans. These groups are also most likely to value conveniences and to snack between meals (47). Recognizing that snacking is a common eating behavior, especially among children and adolescents, the DGA 2020–25 make the case for nutrient-dense snacks (48). Pulse-based snacks are a rapidly growing trend, with the global pulse snack market size estimated at USD 6.8 billion in 2024 (49). According to Pulse Canada (50), 7% of all snack food launches in North America in 2020 contained pulses, including peas, lentils, chickpeas, and beans. Breakfast cereals reformulated to include pea flour of lentil flour had improved nutrient density scores and lowered greenhouse gas emissions (51). Studies have explored methods for producing snacks where cereals or tubers were replaced with at least 50% pulses, allowing for the reformulation of pasta, baked goods, snacks, and protein bars (52). The high fiber content of pulses can support satiety and cardio-metabolic health (53). Pulses are also regarded as environmentally friendly and a vital component of regenerative agriculture (54).

## 5 Conclusion

Despite being recognized as nutritious, sustainable, and affordable sources of plant-based protein, pulses remain largely under-consumed in the US (55). While pulse consumers, especially Mexican-Americans and lower-income groups, approached the recommended 2.5 cups/week, population-wide consumption was closer to 0.68 cups/week. Implementing DGA 2025 will require targeted promotion strategies by pulse type directed at specific population groups.

## Data Availability

Publicly available datasets were analyzed in this study. These data can be found at: https://wwwn.cdc.gov/nchs/nhanes/ (Accessed 2025 May 29). Nutrient composition data are found at: https://www.ars.usda.gov/ARSUserFiles/80400530/pdf/fndds/FNDDS_2017_2018_factsheet.pdf (Accessed 2025 May 14).
