# Supplementary material for: Pulse consumption trends in the US in the context of 2025–30 Dietary Guidelines for Americans: analyses of NHANES data for 1999–2018
Source: Front Nutr. 2025 Aug 21;12:1638519. doi: 10.3389/fnut.2025.1638519 (PMC12408265; doi:10.3389/fnut.2025.1638519)
Supplement: Supplementary file 1 [file Table_1.docx]

**SUPPLEMENTAL TABLES**

**Pulse consumption trends in the US in the context of 2025-30 Dietary Guidelines for Americans:: Analyses of NHANES data for 1999-2018**

**Adam Drewnowski, Catherine Zavela, Vanessa Tran, Greeshma Mallya, Zach Conrad**

SUPPLEMENTAL Table 1 (Proportion of adults who consumed pulses on a given day by race/ethnicity) 1999-2018 (n=48,738)

| Year |  | All |  | | Mexican Americans | |  | Non Hispanic Black | | |  | Other/Multiracial | |  | non Hispanic White | |
| --- | --- | --- | --- | --- | --- | --- | --- | --- | --- | --- | --- | --- | --- | --- | --- | --- |
|  |  | Mean | CI | | Mean | SEM |  | | Mean | SEM |  | Mean | SEM |  | Mean | SEM |
| 1999-2000 |  | 0.182 | (0.162-0.202) | | 0.381 | (0.342-0.420) |  | | 0.126 | (0.104-0.148) |  | 0.239 | (0.180-0.298) |  | 0.16 | (0.140-0.180) |
| 2001-2002 |  | 0.174 | (0.154-0.194) | | 0.37 | (0.311-0.429) |  | | 0.149 | (0.092-0.206) |  | 0.235 | (0.176-0.294) |  | 0.151 | (0.131-0.171) |
| 2003-2004 |  | 0.163 | (0.143-0.183) | | 0.314 | (0.275-0.353) |  | | 0.098 | (0.074-0.122) |  | 0.153 | (0.114-0.192) |  | 0.158 | (0.138-0.178) |
| 2005-2006 |  | 0.153 | (0.133-0.173) | | 0.333 | (0.294-0.372) |  | | 0.123 | (0.090-0.156) |  | 0.163 | (0.104-0.222) |  | 0.137 | (0.117-0.157) |
| 2007-2008 |  | 0.161 | (0.141-0.181) | | 0.335 | (0.296-0.374) |  | | 0.104 | (0.082-0.126) |  | 0.189 | (0.150-0.228) |  | 0.145 | (0.106-0.184) |
| 2009-2010 |  | 0.154 | (0.134-0.174) | | 0.308 | (0.288-0.328) |  | | 0.109 | (0.080-0.138) |  | 0.187 | (0.167-0.207) |  | 0.137 | (0.117-0.157) |
| 2011-2012 |  | 0.189 | (0.169-0.209) | | 0.272 | (0.213-0.331) |  | | 0.116 | (0.100-0.132) |  | 0.254 | (0.215-0.293) |  | 0.179 | (0.159-0.199) |
| 2013-2014 |  | 0.173 | (0.153-0.193) | | 0.358 | (0.280-0.436) |  | | 0.137 | (0.104-0.170) |  | 0.234 | (0.195-0.273) |  | 0.141 | (0.121-0.161) |
| 2015-2016 |  | 0.195 | (0.175-0.215) | | 0.341 | (0.302-0.380) |  | | 0.122 | (0.093-0.151) |  | 0.229 | (0.190-0.268) |  | 0.179 | (0.140-0.218) |
| 2017-2018 |  | 0.177 | (0.157-0.197) | | 0.317 | (0.278-0.356) |  | | 0.116 | (0.092-0.140) |  | 0.235 | (0.176-0.294) |  | 0.151 | (0.131-0.171) |
| Mean |  | 0.172 | (0.163-0.181) | | 0.333 | (0.313-0.353) |  | 0.120 | | (0.111-0.129) |  | 0.212 | (0.190-0.234) |  | 0.154 | (0.144-0.163) |
| Trend, β |  |  | 0.03 | |  | -0.01 |  |  | | 0.00 |  |  | 0.003 |  |  | 0.003 |
| Trend, P |  |  | 0.154 | | 0.40 | |  | 0.903 | | |  | 0.81 | |  | 0.414 | |
|  |  |  |  |  |  |  |  |  | |  |  |  |  |  |  |  |
|  | | |  | |  |  |  |  | |  |  |  |  |  |  |  |

| Supplemental Table 2: Mean per capita intake of pulses in oz/day among all US adults, 1999-2018 (n=48,738) | | | | | | | | | | | | | | | |
| --- | --- | --- | --- | --- | --- | --- | --- | --- | --- | --- | --- | --- | --- | --- | --- |
| Year |  | All pulses | |  | Beans | |  | Chickpeas | |  | Peas | |  | Lentils | |
|  |  | Mean oz. (95% CI)^1^ | | | | | | | | | | | | | |
| 1999-2000 |  | 0.43 | (0.36-0.51) | | 0.38 | (0.32-0.44) |  | 0.01 | (0-0.02) |  | 0.01 | (0-0.02) |  | 0.03 | (-0.01-0.07) |
| 2001-2002 |  | 0.41 | (0.35-0.47) | | 0.38 | (0.33-0.43) |  | 0.01 | (0.01-0.02) |  | 0.00 | (0-0.01) |  | 0.01 | (0.01-0.02) |
| 2003-2004 |  | 0.32 | (0.26-0.37) | | 0.28 | (0.23-0.34) |  | 0.01 | (0-0.02) |  | 0.00 | (0-0.01) |  | 0.02 | (0.01-0.03) |
| 2005-2006 |  | 0.33 | (0.28-0.39) | | 0.31 | (0.25-0.36) |  | 0.01 | (0.01-0.02) |  | 0.01 | (0-0.02) |  | 0.01 | (0-0.01) |
| 2007-2008 |  | 0.36 | (0.29-0.43) | | 0.32 | (0.25-0.38) |  | 0.01 | (0-0.02) |  | 0.01 | (0-0.01) |  | 0.02 | (0.01-0.04) |
| 2009-2010 |  | 0.35 | (0.3-0.4) |  | 0.29 | (0.25-0.34) |  | 0.02 | (0.01-0.03) |  | 0.01 | (0-0.01) |  | 0.03 | (0.01-0.04) |
| 2011-2012 |  | 0.42 | (0.36-0.49) | | 0.35 | (0.29-0.42) |  | 0.03 | (0.02-0.05) |  | 0.00 | (0-0.01) |  | 0.03 | (0.02-0.04) |
| 2013-2014 |  | 0.41 | (0.37-0.45) | | 0.34 | (0.3-0.38) |  | 0.02 | (0.01-0.03) |  | 0.00 | (0-0.01) |  | 0.03 | (0.02-0.04) |
| 2015-2016 |  | 0.45 | (0.37-0.52) | | 0.39 | (0.31-0.47) |  | 0.02 | (0.01-0.03) |  | 0.01 | (0-0.01) |  | 0.03 | (0.02-0.04) |
| 2017-2018 |  | 0.42 | (0.34-0.5) |  | 0.35 | (0.28-0.42) |  | 0.03 | (0.02-0.04) |  | 0.00 | (0-0.01) |  | 0.04 | (0.02-0.06) |
| Mean |  | 0.39 | (0.37-0.41) | | 0.34 | (0.32-0.36) |  | 0.02 | (0.02-0.02) |  | 0.01 | (0-0.01) |  | 0.02 | (0.02-0.03) |
| Trend, β |  | 0.01 | (0-0.01) |  | 0.00 | (-0.01-0.01) | | 0.00 | (0-0) |  | 0.00 | (0-0) |  | 0.00 | (0-0) |
| Trend, P |  | 0.126 | |  | 0.599 | |  | <0.001 | |  | 0.715 | |  | 0.092 | |
|  |  |  |  |  |  |  |  |  |  |  |  |  |  |  |  |
|  | | | |  |  |  |  |  |  |  |  |  |  |  |  |

| Supplemental Table 3: Mean per capita intake of pulses in oz/day among US adults, by age group, 1999-2018 (n=48,738) | | | | | | | | | | | | |
| --- | --- | --- | --- | --- | --- | --- | --- | --- | --- | --- | --- | --- |
| Year |  | 20-30 y | |  | 31-50 y | |  | 51-70 y | |  | 70+ y | |
|  |  | Mean oz. (95% CI)^1^ | | | | | | | | | | |
| 1999-2000 |  | 0.38 | (0.29-0.48) |  | 0.47 | (0.34-0.61) |  | 0.45 | (0.32-0.58) |  | 0.38 | (0.3-0.45) |
| 2001-2002 |  | 0.43 | (0.32-0.55) |  | 0.44 | (0.35-0.52) |  | 0.41 | (0.3-0.51) |  | 0.29 | (0.22-0.36) |
| 2003-2004 |  | 0.39 | (0.28-0.49) |  | 0.33 | (0.24-0.43) |  | 0.27 | (0.22-0.31) |  | 0.26 | (0.19-0.34) |
| 2005-2006 |  | 0.34 | (0.24-0.44) |  | 0.34 | (0.27-0.42) |  | 0.36 | (0.28-0.44) |  | 0.21 | (0.11-0.3) |
| 2007-2008 |  | 0.33 | (0.25-0.41) |  | 0.41 | (0.29-0.52) |  | 0.35 | (0.26-0.43) |  | 0.28 | (0.16-0.41) |
| 2009-2010 |  | 0.36 | (0.28-0.45) |  | 0.39 | (0.33-0.46) |  | 0.31 | (0.23-0.38) |  | 0.27 | (0.2-0.34) |
| 2011-2012 |  | 0.35 | (0.28-0.42) |  | 0.46 | (0.35-0.57) |  | 0.45 | (0.37-0.54) |  | 0.35 | (0.23-0.47) |
| 2013-2014 |  | 0.37 | (0.31-0.42) |  | 0.48 | (0.4-0.57) |  | 0.37 | (0.3-0.45) |  | 0.32 | (0.22-0.43) |
| 2015-2016 |  | 0.39 | (0.27-0.5) |  | 0.52 | (0.42-0.62) |  | 0.43 | (0.31-0.56) |  | 0.37 | (0.26-0.48) |
| 2017-2018 |  | 0.43 | (0.32-0.54) |  | 0.41 | (0.33-0.49) |  | 0.44 | (0.26-0.61) |  | 0.36 | (0.25-0.48) |
| Trend, β |  | 0.00 | (-0.01-0.01) | | 0.01 | (0-0.02) |  | 0.01 | (-0.01-0.02) | | 0.01 | (0-0.02) |
| Trend, P |  | 0.883 | |  | 0.203 | |  | 0.360 | |  | 0.117 | |
|  |  |  |  |  |  |  |  |  |  |  |  |  |
| . | | | |  |  |  |  |  |  |  |  |  |

Supplemental Table 4: Mean per capita intake of pulses **among all US adults**, by sex, income-to-poverty ratio (IPR) and education level, 1999-2018 (n=44,609)

| Year |  | Male | |  | Female | | IPR 0.00-1.85 | |  | IPR 1.86+ | |  | | Less than college | | | Some college | |  |
| --- | --- | --- | --- | --- | --- | --- | --- | --- | --- | --- | --- | --- | --- | --- | --- | --- | --- | --- | --- |
|  |  | Mean oz. (95% CI)^1^ | | | | | Mean oz. (95% CI)^1^ | | | | | |  | | Mean oz. (95% CI)^1^ | | | | |
| 1999-2000 |  | 0.47 | (0.38-0.56) |  | 0.40 | (0.31-0.5) | 0.45 | (0.33-0.57) |  | 0.40 | (0.32-0.49) |  | | 0.49 | | (0.39-0.6) | 0.37 | (0.3-0.44) |  |
| 2001-2002 |  | 0.48 | (0.4-0.56) |  | 0.34 | (0.29-0.39) | 0.56 | (0.47-0.65) |  | 0.34 | (0.28-0.41) |  | | 0.52 | | (0.45-0.59) | 0.31 | (0.24-0.39) |  |
| 2003-2004 |  | 0.38 | (0.31-0.46) |  | 0.25 | (0.2-0.31) | 0.35 | (0.26-0.43) |  | 0.31 | (0.24-0.37) |  | | 0.36 | | (0.28-0.44) | 0.28 | (0.22-0.35) |  |
| 2005-2006 |  | 0.38 | (0.32-0.44) |  | 0.29 | (0.23-0.35) | 0.44 | (0.34-0.53) |  | 0.30 | (0.24-0.36) |  | | 0.37 | | (0.28-0.46) | 0.30 | (0.24-0.37) |  |
| 2007-2008 |  | 0.43 | (0.34-0.52) |  | 0.30 | (0.23-0.36) | 0.43 | (0.3-0.55) |  | 0.33 | (0.27-0.39) |  | | 0.37 | | (0.27-0.48) | 0.35 | (0.28-0.41) |  |
| 2009-2010 |  | 0.39 | (0.31-0.46) |  | 0.31 | (0.27-0.36) | 0.38 | (0.3-0.46) |  | 0.31 | (0.25-0.38) |  | | 0.40 | | (0.31-0.5) | 0.31 | (0.26-0.36) |  |
| 2011-2012 |  | 0.49 | (0.41-0.56) |  | 0.36 | (0.3-0.43) | 0.46 | (0.34-0.58) |  | 0.39 | (0.33-0.45) |  | | 0.44 | | (0.36-0.52) | 0.41 | (0.33-0.5) |  |
| 2013-2014 |  | 0.47 | (0.42-0.52) |  | 0.34 | (0.31-0.38) | 0.50 | (0.42-0.57) |  | 0.36 | (0.3-0.41) |  | | 0.48 | | (0.39-0.56) | 0.36 | (0.32-0.41) |  |
| 2015-2016 |  | 0.50 | (0.39-0.6) |  | 0.40 | (0.34-0.47) | 0.53 | (0.38-0.69) |  | 0.39 | (0.32-0.47) |  | | 0.55 | | (0.43-0.67) | 0.40 | (0.32-0.47) |  |
| 2017-2018 |  | 0.46 | (0.38-0.54) |  | 0.39 | (0.29-0.48) | 0.46 | (0.37-0.56) |  | 0.35 | (0.28-0.43) |  | | 0.47 | | (0.36-0.59) | 0.39 | (0.32-0.46) |  |
| MEAN |  | 0.45 | (0.42-0.47) |  | 0.34 | (0.32-0.36) | 0.46 | (0.42-0.49) |  | 0.35 | (0.33-0.37) |  | | 0.45 | | (0.42-0.48) | 0.35 | (0.33-0.37) |  |
| Trend, β |  | 0.01 | (0-0.01) |  | 0.01 | (0-0.01) | 0.00 | (-0.01-0.02) |  | 0.00 | (0-0.01) |  | | 0.00 | | (-0.01-0.02) | 0.01 | (0-0.02) |  |
| Trend, P |  |  | 0.278 |  |  | 0.122 |  | 0.446 |  |  | 0.401 |  | |  | | 0.449 |  | 0.017 |  |

Supplemental Table 5: Mean per capita intake of pulses **among all US adults**, by race-ethnicity, 1999-2018 (n=48,738)

| Year |  | Non-Hispanic White | |  | Non-Hispanic Black | |  | Mexican-American | | |  | Other^1^ | |
| --- | --- | --- | --- | --- | --- | --- | --- | --- | --- | --- | --- | --- | --- |
|  |  | Mean oz. (95% CI)^2^ | | | | | | | | | | | |
| 1999-2000 |  | 0.32 | (0.24-0.39) |  | 0.30 | (0.22-0.39) |  | 1.21 | | (0.98-1.43) |  | 0.78 | (0.46-1.1) |
| 2001-2002 |  | 0.31 | (0.25-0.37) |  | 0.41 | (0.27-0.54) |  | 1.05 | | (0.9-1.2) |  | 0.69 | (0.45-0.93) |
| 2003-2004 |  | 0.28 | (0.21-0.34) |  | 0.20 | (0.12-0.27) |  | 0.79 | | (0.63-0.96) |  | 0.36 | (0.21-0.52) |
| 2005-2006 |  | 0.27 | (0.22-0.32) |  | 0.26 | (0.21-0.31) |  | 0.97 | | (0.77-1.17) |  | 0.41 | (0.23-0.6) |
| 2007-2008 |  | 0.31 | (0.21-0.4) |  | 0.22 | (0.17-0.27) |  | 0.87 | | (0.73-1.01) |  | 0.47 | (0.37-0.56) |
| 2009-2010 |  | 0.27 | (0.21-0.33) |  | 0.29 | (0.19-0.39) |  | 0.93 | | (0.78-1.09) |  | 0.44 | (0.37-0.51) |
| 2011-2012 |  | 0.36 | (0.3-0.42) |  | 0.26 | (0.19-0.33) |  | 0.84 | | (0.67-1.02) |  | 0.62 | (0.48-0.76) |
| 2013-2014 |  | 0.28 | (0.23-0.33) |  | 0.37 | (0.28-0.46) |  | 1.07 | | (0.82-1.32) |  | 0.61 | (0.49-0.73) |
| 2015-2016 |  | 0.37 | (0.27-0.47) |  | 0.29 | (0.2-0.38) |  | 0.90 | | (0.81-0.99) |  | 0.61 | (0.49-0.73) |
| 2017-2018 |  | 0.30 | (0.23-0.37) |  | 0.29 | (0.22-0.36) |  | 0.95 | | (0.67-1.24) |  | 0.66 | (0.51-0.82) |
| Mean |  | 0.30 | (0.28-0.33) |  | 0.28 | (0.26-0.31) |  | 0.95 | | (0.89-1.01) |  | 0.58 | (0.53-0.64) |
| Trend, β |  | 0.00 | (0-0.01) |  | 0.00 | (-0.01-0.01) | | | -0.01 | (-0.03-0.01) |  | 0.00 | (-0.02-0.02) |
| Trend, P |  | 0.415 | |  | 0.903 | |  | 0.397 | | |  | 0.811 | |
|  |  |  |  |  |  |  |  |  | |  |  |  |  |
| ^1^Includes other Hispanic, non-Hispanic Asian, and multi-racial. | | | | | | | | | |  |  |  |  |

Supplemental Table 6: Mean per capita intake of pulses among pulse consuming US adults, 1999-2018 (n=9186)

| Year |  | All pulses | |  | Beans | |  | Chickpeas | |  | Peas | |  | Lentils | |
| --- | --- | --- | --- | --- | --- | --- | --- | --- | --- | --- | --- | --- | --- | --- | --- |
|  |  | Mean oz. (CI) | | | | | | | | | | | | | |
| 1999-2000 |  | 2.39 | (2.16-2.62) | | 2.11 | (1.93-2.29) |  | 0.07 | (0.02-0.12) |  | 0.05 | (0.02-0.08) |  | 0.16 | (-0.04-0.36) |
| 2001-2002 |  | 2.37 | (2.19-2.54) | | 2.2 | (2.02-2.38) |  | 0.06 | (0.04-0.09) |  | 0.02 | (0-0.04) |  | 0.08 | (0.04-0.12) |
| 2003-2004 |  | 1.96 | (1.81-2.11) | | 1.76 | (1.6-1.92) |  | 0.06 | (0.03-0.1) |  | 0.01 | (-0.01-0.03) |  | 0.12 | (0.04-0.2) |
| 2005-2006 |  | 2.19 | (2.02-2.37) | | 2.02 | (1.82-2.23) |  | 0.07 | (0.04-0.1) |  | 0.05 | (0-0.1) |  | 0.05 | (0.01-0.09) |
| 2007-2008 |  | 2.21 | (1.96-2.46) | | 1.95 | (1.71-2.18) |  | 0.08 | (0.03-0.13) |  | 0.04 | (0.01-0.07) |  | 0.14 | (0.06-0.23) |
| 2009-2010 |  | 2.24 | (2.09-2.39) | | 1.9 | (1.71-2.08) |  | 0.14 | (0.08-0.2) |  | 0.04 | (0.02-0.08) |  | 0.16 | (0.1-0.22) |
| 2011-2012 |  | 2.25 | (2.06-2.44) | | 1.88 | (1.67-2.1) |  | 0.17 | (0.08-0.27) |  | 0.02 | (0.01-0.04) |  | 0.17 | (0.11-0.24) |
| 2013-2014 |  | 2.33 | (2.19-2.48) | | 1.98 | (1.8-2.15) |  | 0.14 | (0.09-0.19) |  | 0.03 | (0.01-0.04) |  | 0.19 | (0.12-0.26) |
| 2015-2016 |  | 2.27 | (2.02-2.52) | | 2.00 | (1.7-2.3) |  | 0.11 | (0.05-0.17) |  | 0.03 | (0.01-0.06) |  | 0.13 | (0.08-0.18) |
| 2017-2018 |  | 2.38 | (2.14-2.62) | | 1.98 | (1.76-2.21) |  | 0.15 | (0.09-0.21) |  | 0.03 | (0-0.06) |  | 0.22 | (0.1-0.35) |
| Mean |  | 2.26 | (2.19-2.33) | | 1.98 | (1.91-2.05) |  | 0.11 | (0.09-0.13) |  | 0.03 | (0.02-0.04) |  | 0.14 | (0.12-0.17) |
| Trend, β |  |  | 0.00 | |  | -0.01 | |  | 0.01 |  |  | -0.00 |  |  | 0.00 |
| Trend, P |  | 0.50 | |  | 0.37 | |  | 0.000 | |  | 0.54 | |  | 0.14 | |
|  |  |  |  |  |  |  |  |  |  |  |  |  |  |  |  |
| ^1^Unless otherwise noted. | | | |  |  |  |  |  |  |  |  |  |  |  |  |

Supplemental Table 7: Mean per capita intake of pulses among pulse consuming US adults, by sex and income-to-poverty ratio, 1999-2018

| Year |  | Male | |  | Female | | IPR 0.00-1.99 | |  | IPR 2.00+ | |  | Edu cat 1 | | Edu cat 2 | |
| --- | --- | --- | --- | --- | --- | --- | --- | --- | --- | --- | --- | --- | --- | --- | --- | --- |
|  |  | Mean oz. (CI)^1^ | | | | | Mean oz. (CI)^1^ | | | | | | | | | |
| 1999-2000 |  | 2.52 | (2.28-2.76) |  | 2.27 | (1.87-2.67) | 2.50 | (2.14-2.86) |  | 2.19 | (1.93-2.45) |  | 2.55 | (2.23-2.87) | 2.19 | (1.89-2.49) |
| 2001-2002 |  | 2.67 | (2.45-2.89) |  | 2.07 | (1.87-2.27) | 3.03 | (2.73-3.33) |  | 2.04 | (1.78-2.30) |  | 2.76 | (2.54-2.98) | 1.98 | (1.74-2.22) |
| 2003-2004 |  | 2.2 | (2.00-2.40) |  | 1.71 | (1.51-1.91) | 2.28 | (2.02-2.54) |  | 1.81 | (1.65-1.97) |  | 2.23 | (1.97-2.49) | 1.75 | (1.55-1.95) |
| 2005-2006 |  | 2.51 | (2.27-2.75) |  | 1.89 | (1.65-2.13) | 2.49 | (2.19-2.79) |  | 2.06 | (1.86-2.26) |  | 2.37 | (2.13-2.61) | 2.06 | (1.88-2.24) |
| 2007-2008 |  | 2.32 | (2.06-2.58) |  | 2.08 | (1.76-2.40) | 2.6 | (2.26-2.94) |  | 2.03 | (1.79-2.27) |  | 2.59 | (2.29-2.89) | 1.95 | (1.69-2.21) |
| 2009-2010 |  | 2.47 | (2.19-2.75) |  | 2 | (1.86-2.14) | 2.62 | (2.46-2.78) |  | 1.98 | (1.74-2.22) |  | 2.57 | (2.31-2.83) | 2.01 | (1.81-2.21) |
| 2011-2012 |  | 2.36 | (2.16-2.56) |  | 2.13 | (1.89-2.37) | 2.47 | (2.25-2.69) |  | 2.07 | (1.81-2.33) |  | 2.47 | (2.21-2.73) | 2.13 | (1.89-2.37) |
| 2013-2014 |  | 2.44 | (2.28-2.60) |  | 2.21 | (1.97-2.45) | 2.82 | (2.44-3.20) |  | 2.03 | (1.87-2.19) |  | 2.74 | (2.52-2.96) | 2.08 | (1.96-2.20) |
| 2015-2016 |  | 2.47 | (2.07-2.87) |  | 2.08 | (1.86-2.30) | 2.68 | (2.10-3.26) |  | 2.04 | (1.78-2.30) |  | 2.75 | (2.45-3.05) | 2.01 | (1.73-2.29) |
| 2017-2018 |  | 2.57 | (2.29-2.85) |  | 2.21 | (1.91-2.51) | 2.84 | (2.58-3.10) |  | 2 | (1.70-2.30) |  | 2.78 | (2.48-3.08) | 2.15 | (1.89-2.41) |
| Mean |  | 2.45 | (2.37-2.53) |  | 2.07 | (1.99-2.15) | 2.64 | (2.52-2.76) |  | 2.02 | (1.94-2.10) |  | 2.59 | (2.51-2.67) | 2.04 | (1.96-2.12) |
| Trend, β |  |  | -0.00 |  |  | 0.01 |  | 0.02 |  |  | -0.00 |  |  | 0.026 |  | 0.01 |
| Trend, P |  | 0.98 | |  | 0.32 | | 0.42 | |  | 0.87 | |  | 0.09 | | 0.33 | |

Supplemental Table 8: Mean per capita intake of pulses among pulse consuming US adults, by race/ethnicity, 1999-2018 (n=5,895)

| Year |  | Mexican American | |  | Other | | Non Hispanic Black | |  | non Hispanic White | |  | |  |
| --- | --- | --- | --- | --- | --- | --- | --- | --- | --- | --- | --- | --- | --- | --- |
|  |  | Mean oz. (CI)^1^ | | | | | | | | | | |  | |
| 1999-2000 |  | 3.11 | (2.61-3.61) |  | 3.25 | (2.61-3.89) | 2.35 | (1.81-2.89) |  | 1.99 | (2.24-2.56) |  | |  |
| 2001-2002 |  | 2.85 | (2.55-3.15) |  | 2.94 | (2.18-3.70) | 2.I77 | (2.39-3.15) |  | 2.09 | (1.93-2.25) |  | |  |
| 2003-2004 |  | 2.61 | (2.27-2.95) |  | 2.4 | (1.82-2.98) | 2.01 | (1.39-2.63) |  | 1.77 | (1.57-1.97) |  | |  |
| 2005-2006 |  | 2.91 | (2.29-3.53) |  | 2.53 | (1.99-3.07) | 2.11 | (1.73-2.49) |  | 1.97 | (1.79-2.15) |  | |  |
| 2007-2008 |  | 2.55 | (2.19-2.91) |  | 2.41 | (2.13-2.69) | 2.07 | (1.79-2.35) |  | 2.09 | (1.71-2.47) |  | |  |
| 2009-2010 |  | 2.99 | (2.61-3.37) |  | 2.35 | (2.09-2.61) | 2.64 | (2.18-3.10) |  | 1.96 | (1.70-2.22) |  | |  |
| 2011-2012 |  | 3.15 | (2.87-3.43) |  | 2.45 | (2.13-2.77) | 2.25 | (1.69-2.81) |  | 2.02 | (1.80-2.24) |  | |  |
| 2013-2014 |  | 3.01 | (2.47-3.55) |  | 2.61 | (2.31-2.91) | 2.74 | (2.36-3.12) |  | 1.93 | (1.83-2.03) |  | |  |
| 2015-2016 |  | 2.65 | (2.43-2.87) |  | 2.65 | (2.45-2.85) | 2.32 | (1.76-2.88) |  | 2.05 | (1.61-2.49) |  | |  |
| 2017-2018 |  | 3 | (2.36-3.64) |  | 2.85 | (2.63-3.07) | 2.49 | (2.03-2.95) |  | 1.97 | (1.71-2.23) |  | |  |
| MEAN |  | 2.88 | (2.74-3.02) |  | 2.67 | (2.53-2.81) | 2.4 | (2.24-2.56) |  | 1.99 | (1.91-2.07) |  | |  |
| Trend, β |  |  | 0.00 |  |  | -0.02 |  | 0.01 |  |  | 0.00 |  | |  |
| Trend, P |  | 0.89 | |  | 0.42 | | 0.60 | |  | 0.81 | |  | |  |
